# A Critical Reappraisal of Haloperidol for Delirium Management in the Intensive Care Unit: Perspective from Psychiatry

**DOI:** 10.3390/jcm14020438

**Published:** 2025-01-11

**Authors:** Shixie Jiang, Matthew Gunther

**Affiliations:** 1 Department of Psychiatry, University of Florida College of Medicine, Gainesville, FL 32608, USA; 2 Department of Psychiatry and Behavioral Sciences, School of Medicine, Stanford University, Stanford, CA 94305, USA; guntherm@stanford.edu

**Keywords:** delirium, encephalopathy, acute brain failure, antipsychotics, haloperidol, dopamine, psychopharmacology, intensive care unit, critical care

## Abstract

Delirium is a complex neuropsychiatric syndrome with multifactorial pathophysiology, encompassing a wide range of neuropsychiatric symptoms, and its management remains a significant challenge in critical care. Although often managed with antipsychotics, like haloperidol, current research has predominantly focused on dopamine dysregulation as the primary driver of delirium, overlooking its broader neuroanatomical and neurochemical underpinnings. This has led to a majority of research focusing on haloperidol as a treatment for intensive care unit (ICU) delirium. Our review critically evaluates the role of haloperidol in ICU delirium management, particularly in light of recent large-scale randomized controlled trials (RCTs) that have primarily focused on delirium-free days and mortality as the primary endpoints. These studies highlight an limited understanding of the true nature of delirium treatment, which requires a broader, neuropsychiatric approach. We argue that future research should shift focus to neuropsychiatric symptoms such as agitation and psychosis and explore the clinical and functional benefits of reducing these distressing symptoms. Additionally, the stratification of delirium by subtypes and etiology, the enhancement of detection tools, and the adoption of multi-intervention and multi-disciplinary care approaches should be prioritized. Despite the methodological flaws in these studies, the findings support the safety of haloperidol in the ICU setting, with minimal risk of adverse events, particularly cardiac and neuropsychiatric. Moving forward, delirium research must integrate modern neuroscientific understanding and adopt more multi-disciplinary input and nuanced, patient-centered approaches to truly advance clinical care and outcomes.

## 1. Introduction

Delirium is a neuropsychiatric syndrome precipitated by a medical insult and characterized by an acute or subacute onset and fluctuating course, with disruptions in attention, arousal, cognitive domains, psychomotor state, perceptions, and emotions [1]. It is exceedingly common in the intensive care unit (ICU) setting and may occur in up to 84% of mechanically ventilated patients [2,3]. It incurs an immense stress on patients, their families, and caregivers and is associated with a greater risk of institutionalization, mortality, and long-term cognitive and psychiatric sequelae [4,5,6,7]. Despite such an enormous public health burden, no single clinical intervention has been shown to definitively treat delirium besides correcting the underlying medical problem(s) [1]. Pharmacologically, many classes of medications have been trialed for the treatment of delirium with variable success [8]. Antipsychotics, historically used by psychiatrists to treat psychotic and mood disorders, have been extensively studied for delirium because of the similar clinical presentations, as delirious patients often exhibit psychosis, impulsivity, and emotional disturbances [9]. The most well-known and routinely used antipsychotic for this purpose is haloperidol.

Haloperidol is a first-generation antipsychotic synthesized by Janssen Pharmaceuticals in 1958 [10]. At the American Psychiatric Association Annual Meeting in 1978, consultation-liaison psychiatrists were the first to present their experience using intravenous haloperidol for the management of delirium-related agitation [11]. In every case, amelioration of agitation was rapidly achieved to allow for planned diagnostic or therapeutic interventions. Since then, haloperidol has remained the first-line pharmacological agent for the management of delirium for many medical providers, likely due to its favorable pharmacokinetic and receptor profiles. It has a high binding affinity for dopamine (D2) receptors with a 14-hour half-life, thus producing a long-lasting effect [12]. Its additional lack of any serotonergic, alpha, histamine, or cholinergic receptor profiles makes it attractive for not provoking drug–drug interactions or antagonistic effects in the setting of delirium [13].

Despite its traditionally widespread use, recent clinical trial results have led to concerns regarding its role in the management of delirium. Consecutive, large, double-blind, placebo-controlled, randomized trials reported that the use of haloperidol did not beneficially alter the duration of delirium or mortality rates [14,15]. As such, authors have urged caution in the subsequent delirium literature and clinical practice, suggesting that haloperidol should not be routinely used in the management of ICU delirium [16]. Given that this directly contrasts with the perspective of psychiatrists, who have historically been the foremost experts in both the diagnosis and management of delirium, as well as in the use of antipsychotics for neuropsychiatric disorders, we sought to provide an updated critique of haloperidol’s use in delirium. In this review, we will first summarize the pathophysiology of ICU delirium and explain how the hypothesis that a selective dopamine-focused antipsychotic like haloperidol could putatively “treat” delirium is fundamentally flawed. Thereafter, we will analyze the most salient and robust studies conducted on haloperidol for delirium and then discuss methodological problems due to misunderstanding the purpose of antipsychotics in delirium. Finally, we will suggest future directions in order to move forward and eliminate the confusion surrounding haloperidol and antipsychotics so that we may all work together to truly progress the management of ICU delirium.

## 2. Delirium in the Intensive Care Setting: Pathophysiology and Contribution from Neurotransmitters

The pathophysiology behind the development of delirium is multifactorial, involving disruption in neurotransmission as well as neural network dysconnectivity [17,18]. Pathophysiology can vary depending on the etiology of delirium (e.g., sepsis, traumatic brain injury). Inherent to the development of delirium is the patient’s inherent risk profile, determined by examining multiple patient factors, including the risk conferred by the patient’s underlying central nervous system (CNS) substrate (e.g., cognitive impairment) and the addition of acute precipitating factors (e.g., inflammation, infection) [1,17,18,19]. Patients in the intensive care setting represent one of the highest-risk populations due to the unique combination of predisposing vulnerabilities and multiple precipitating and perpetuating factors [2,20]. The downstream consequences of prolonged delirium in the intensive care setting can be particularly devastating, with sequelae including the development of neurocognitive disorders, post-traumatic stress disorders, and other psychopathologies [21].

**Figure 1:**
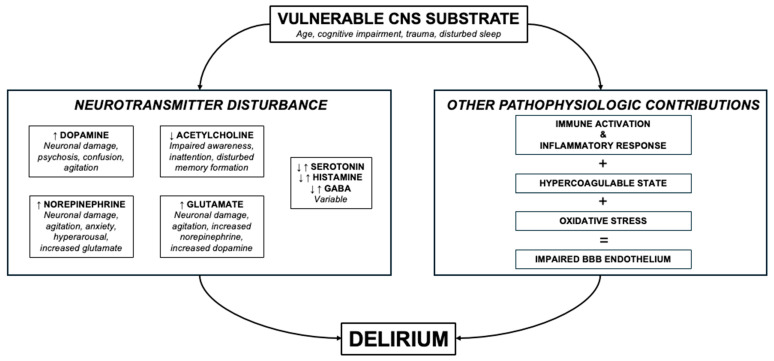
Brief overview of ICU delirium pathophysiology. Abbreviations: CNS = central nervous system; GABA = gamma-aminobutyric acid; BBB = blood–brain barrier.

Disruption in neurotransmission is one of the key factors described in the literature surrounding delirium pathophysiology (Figure 1) [22]. Deficiency in acetylcholine is one of the primary disturbances, supported by the evidence that medications with anticholinergic burden worsen mental status, precipitate delirium, and increase the severity of delirium [17,18,22]. Dopamine dysregulation is another common neurotransmitter implicated in delirium, often thought to be a primary contributor to the neurobehavioral symptoms of delirium [17,18,22,23]. Increased availability of glutamate and norepinephrine, decreased availability of melatonin, and variable levels of gamma-aminobutyric acid (GABA), serotonin, and histamine all contribute to delirium pathophysiology as well, with the level of disturbance being dependent on the pathophysiologic etiology [1,17]. The complex interplay between these neurotransmitters results in the ability for delirium to masquerade as virtually any psychiatric disorder, including psychosis, anxiety, and depression. Additionally, this multifactorial impact on CNS function supports the reality that no single psychotropic agent will ever be able to “treat” delirium by addressing these disturbances.

A deficit in cholinergic function (acutely and with contributions from more chronic conditions like cognitive impairment or dementia) is a leading hypothesis in delirium development [22,24]. Acetylcholine plays a key role in awareness and attention, both of which are disturbed in delirium. It is essential for memory formation and sleep regulation, with disruption leading to other neurotransmitter disturbances (such as dopamine) [17,25]. Multiple studies have demonstrated the relationship between low acetylcholine levels and the development of delirium, including from pathophysiologic conditions with low cholinergic states (such as sepsis) or the combined anticholinergic burden of medications [17,24,26,27]. Patients in the intensive care setting are at higher risk for impaired cholinergic transmission, with studies demonstrating the role of hepatic failure, renal failure, impaired mobility, opioid use, benzodiazepine use, and anesthesia in its development [17,27,28]. This risk is compounded by the known decrease in acetylcholine in the elderly and those with neurocognitive disorders.

Multiple sources of delirium (such as traumatic brain injury or decreased cerebral perfusion secondary to hypoxia) involve a pathophysiologic state of hyperdopaminergic excess; this excess is seen in all motoric subtypes of delirium, especially hyperactive delirium [1,17,23]. Dopamine production is directly enhanced in states of oxidative stress (e.g., sepsis) due to a combination of factors, including increased synthesis [17,29]. Breakdown of dopamine is also impaired in states of hypoxia due to inhibited catechol-o-methyl transferase (COMT) enzymes [17,30]. Lastly, dopamine receptor function may be directly affected, with evidence that genetic polymorphisms or even an increased amount of receptors predispose patients to delirium [23,31]. Mechanistically, neurobehavioral symptoms can be the result of direct dopaminergic effects (akin to toxicity from amphetamine drugs) or through CNS injury via glutamatergic as well as dopaminergic mechanisms [17,23,31].

Patients with delirium face further CNS insults from increased glutamatergic and noradrenergic tones [17,32,33]. Excess glutamate and subsequent NMDA receptor activation contribute to neuronal degeneration and death, mostly through apoptotic mechanisms secondary to calcium influx [17,34]. Increased norepinephrine, often acutely released in the setting of hypoxia or ischemia, leads to further neuronal injury, which also facilitates further glutamate release [17,33]. These neurotransmitter disturbances (dopamine, norepinephrine, and glutamate) are often the source of the neuropsychiatric symptoms of delirium. For example, increases in dopamine or glutamate can lead to psychotic symptoms, while increases in norepinephrine can result in anxiety or other hyperarousal symptoms [1,17,35,36]. Dysregulated neurotransmitters are not acting in isolation; rather, it is the interplay of these neurotransmitters in combination that can lead to delirium. For example, glutamate and dopamine are synergistic in their potential to lead to excitotoxicity, with increased levels of dopamine facilitating glutamatergic neurotoxic injury. Circulating catecholamines, including excess norepinephrine, can further enhance glutamatergic CNS damage. The potential for individual neurotransmitter aberrancies to impact other neurotransmitter systems further adds to the complexity of delirium pathophysiology [17].

Disturbance in neurotransmission is not the only pathway by which delirium develops. Multiple predisposing factors also contribute, including impaired cognitive functioning, other CNS injuries, including trauma, polypharmacy, and sleep disruption [18,19,20,25]. Many of these contributors are non-modifiable, present in ICU populations, and elevate the delirium risk precipitously. Precipitating factors for delirium involve inflammation and oxidative stress, both of which play major roles in the development of ICU delirium specifically [17,19,34]. Metabolic dysfunction, cytokine activation, and pro-inflammatory mediator signaling increase the vulnerability of the brain to insult and subsequent delirium. In sepsis, the hypercoagulable state combined with reduced fibrinolysis both result in microthrombosis, tissue hypoxia, and potential CNS injury [17,34,37]. The total effect of these cascades results in blood–brain barrier permeability, impacting the susceptibility of the CNS to neurotoxins, overall increasing brain inflammation, and further oxygenation deficits, all of which perpetuate brain dysfunction [17,38,39].

Multiple pharmacologic agent classes have been studied to reduce the delirium burden through targeting various neurotransmitter disturbances or through addressing inflammation or other pathophysiologic factors directly [18,40]. Results have, thus far, been limited, primarily due to delirium’s complex pathophysiology that limits the ability for one pharmacologic agent to address the various disturbances seen in patients [1,40]. This is further complicated even by subtypes of delirium etiologies that have irregularities in which neurotransmitter is disturbed and to what degree [1,17]. For example, in alcohol withdrawal, glutamate and norepinephrine are in excess with a good response to pharmacologic interventions consisting, in part, of GABA modulators, anti-glutamatergics, and anti-sympathetics. Alternatively, in hepatic encephalopathy, dopamine and norepinephrine are decreased from baseline with notable excess in GABA; GABA modulators, in these cases, would worsen delirium [17]. Dopamine antagonists (specifically antipsychotics) are primarily used to target the neuropsychiatric sequelae of delirium. Similar to the concept of reducing dopaminergic tone in a patient with amphetamine-driven psychosis, antipsychotics play a similar role in reducing agitation or perceptual disturbance through the pathophysiologic disturbance in delirious patients [18]. Beyond managing the neurobehavioral effects of delirium, however, dopamine antagonists may play a role in addressing the dopaminergic surge as delirium develops, potentially playing a role in delirium prevention [1,15,40,41,42]. As a class, they have been the most studied agents for delirium management, with haloperidol being the most common [15,40,42].

## 3. Methods

There are many studies that aim to evaluate the effectiveness of antipsychotics, most commonly haloperidol, in reducing the impact of delirium. These include a range of types of study, including case reports to randomized controlled trials. Antipsychotics have been studied not only for the management of delirium symptoms but also for their ability to prevent delirium development.

We reviewed all randomized controlled trials evaluating the use of haloperidol versus placebo in mitigating the effects of delirium on patients in the critical care setting. All data were extracted from reviewed studies and are included in this manuscript. We searched PubMed and Web of Science from database inception to 1 November 2024, using MeSH or search terms in the following combinations: “haloperidol” AND (“delirium OR encephalopathy”) AND (“intensive care” OR “critical care”). In the assessment, S.J. and M.G. screened titles and abstracts for inclusion and exclusion criteria. S.J. and M.G. independently reviewed the full text of remaining articles and searched reference lists of eligible manuscripts to identify other possible studies.

The inclusion criteria involved randomized controlled trials (RCTs) set in the intensive care setting where haloperidol was studied versus placebo for the management of delirium. Articles were excluded for the following reasons: studies examining the prevention (rather than the management) of delirium; comparators other than placebo; wrong study design (case reports, cohort studies, systematic reviews, or perspective pieces); pediatric populations only (participants <18 years old); wrong indication for haloperidol (e.g., for nausea instead of delirium); symptom management for non-delirium-related diagnoses (e.g., agitation due to schizophrenia); and non-English articles.

The study characteristics, including lead author, year of study, country, and study design, were collected. Demographic data were extracted to examine population differences and potential bias. In each study, a delirium assessment and management strategies were extracted, including screening tools utilized (if any), haloperidol dosing strategies, availability of rescue medication, and length of study intervention. Lastly, primary and secondary outcomes were examined.

## 4. A Review of Trials Examining Haloperidol Versus Placebo for Delirium Management

Five RCTs were included in our review (Table 1); one trial of the five was terminated early due to difficulty with recruitment (ORIC-I) [14,15,43,44,45]. These examined critically ill adult populations who screened positive for delirium. The exclusionary criteria were similar across groups and included patients with neurocognitive impairment or dementias, contraindications to haloperidol use (e.g., neuroleptic malignant syndrome or QT prolongation), and ongoing antipsychotic use for another indication. Most excluded patients had acute neurologic conditions. Each study sought to examine the effects of scheduled haloperidol, with variability in dosing, on either mortality or the number of delirium- and coma-free days [14,15,43,44,45]. Patients were enrolled and assigned to either intervention (haloperidol) or placebo groups. Haloperidol administration across the studies was typically at fixed amounts, either twice daily or three times a day (with potential for rescue medication (alpha-2 agonists, benzodiazepines, propofol, or other antipsychotics)) [14,15,43,44,45]. Dosing varied widely, with one study noting mean daily dosing (EuRIDICE: 5.6 mg/day (noted as mg/kg)), two studies noting median dosing (Andersen: 8.3 mg/day; Girard: 11 mg/day), and one study reporting a maximum dose only (Garg: 30 mg/day). Patients were assessed for delirium, typically with the Confusion Assessment Method for the Intensive Care Unit (CAM-ICU), often multiple times per day. The results of these assessments guided the interventions, with each study allowing for a dosing adjustment of haloperidol if a patient remained delirious. In the Andersen study, dosing was fixed throughout the intervention period but could be increased as needed for ongoing delirium symptoms. In the Girard and EuRIDICE studies, dosing was doubled based on the delirium screening result alone (see Table 1 for dosing specifics). No adjustments were made for the motoric subtype of delirium. For example, in the trial by Girard and colleagues, patients with hypoactive or hyperactive delirium could receive up to 20 mg of haloperidol in one day [14]. Once a patient was no longer delirious, haloperidol was either held (Andersen) or tapered and then held (Girard; EuRIDICE). Each study allowed for the interventions to be resumed if patients became delirious again [14,15,44].

All four published RCTs were negative in that there was no effect of haloperidol on the primary measures of the number of delirium and coma-free days, nor mortality [14,15,44,45]. These outcomes were selected, given the large body of literature describing the increased duration of ICU stay in patients with delirium, as well as the increased risk of mortality associated with this diagnosis. Secondary outcomes were reported and were mostly focused on the duration of time in the ICU, in the hospital setting, or time until freedom from mechanical ventilation—other important metrics that reflect increased risk of mortality [14,15,44,45]. Secondary outcomes were not significant across the studies, except for some outcomes in the EuRIDICE trial [44]. Beyond the intervention period, nearly all studies examined the mortality rate at 30 days, 90 days, or even 12 months. EuRIDICE, however, was the only study to examine other metrics, including quality of life after hospitalization and the development of post-traumatic stress disorder (both 12 months after hospitalization). All trials examined the adverse events in the study groups, which were not significant, including the potential for QT prolongation with haloperidol use [14,15,42,44,45].

Of the studies, only the EuRIDICE trial examined direct clinical symptoms related to delirium, including the presence of hallucinations/psychosis, subsequent delusional memories, and agitation (evaluated by patient self-extubation, attempts at interfering with medical devices, and attempts to fall/step out of bed) [44]. While not all secondary outcomes in the EuRIDICE trial were significant, the inclusion of these factors is important as all pertain to the rationale that the consulting psychiatrists employ when recommending psychotropic interventions for delirium. The authors of the EuRIDICE trial reported significant reduction in agitation; decreased distress and agitation are key indicators to a consulting psychiatrist that a pharmacologic strategy is working. The authors also reported a reduction in benzodiazepine use and a significant reduction in intrusive memories following hospitalization (but no impact on the incidence of post-traumatic stress disorder in patients who experienced delirium) [44]. There are growing data describing the traumatic experience of an intensive care stay, with psychiatric symptoms exacerbated by intrusive or delusional memories [46,47,48]. The formation of these memories, commonly occurring in delirium, is exacerbated by GABAergic medications such as benzodiazepines [46]. Reduction in benzodiazepine use, facilitated by the utilization of antipsychotic medication instead, has the potential to improve outcomes and distress surrounding one’s medical experience.

## 5. Reappraisal of the Published Literature

The fundamental limitation in published RCTs and meta-is the assumption that haloperidol or any single drug by itself can be used for the “treatment” of delirium. As discussed earlier in this review, the pathophysiology of ICU delirium is extraordinarily heterogeneous. Based on the neurotransmitter hypothesis, dopamine dysregulation is likely implicated in all types of delirium; however, whether it is increased or decreased and how much depends on the etiology and subtype [17]. Additionally, its precise role in the development of neuropsychiatric symptoms outside of aggression, agitation, impulsivity/disinhibition, and psychosis is much less clear. Thus, given the diverse range of symptoms that can present in delirium, the utilization or study of haloperidol alone for the global treatment of the syndrome neglects to address all of the complex neurobiological mechanisms involved. This reductionistic mistake was similarly made nearly seven decades ago in the field of psychiatry when we overemphasized the “dopamine hypothesis” for schizophrenia. Early work strongly proposed that an imbalance in dopamine neurotransmission, particularly hyperdopaminergic processes, existed at the crux of schizophrenic symptoms [49]. While all antipsychotics modulate dopamine activity, psychiatrists have long recognized that dopamine is primarily implicated only in positive symptoms (e.g., psychosis, delusions), with much less involvement in the cognitive and emotional symptoms of the disorder [50]. Moreover, dopamine may play a more indirect role in the overall pathophysiology than previously thought. Modern evidence suggests that other neurotransmitters, such as acetylcholine, serotonin, and glutamate, and secondary messengers may play an even more significant role in the core symptoms of schizophrenia [51].

Without a clear understanding of the underlying pathophysiology, the choice of primary endpoints and the resulting outcomes in the RCTs are understandable. Focusing on delirium-free and coma-free days as primary endpoints oversimplifies the complexity of ICU delirium and may not fully capture its nature. By combining coma and delirium into one endpoint, these studies treat two distinct conditions as if they are interchangeable. While both involve alterations in consciousness, they are fundamentally different phenomena with different neurobiology and clinical implications [52]. Delirium is a fluctuating neuropsychiatric state, whereas coma is a profound, sustained loss of consciousness. These two states may co-occur, but they are not synonymous, and combining them into a single outcome measure is misleading. Moreover, the administration of an antipsychotic with potent sedative properties, like haloperidol, to already comatose patients, with the aim of “treating” delirium via dopamine reduction, is illogical and potentially dangerous for patients. The rationale behind this combined endpoint appears to stem from the difficulty of distinguishing between delirium and coma, particularly when patients are heavily sedated and unable to participate in assessments. In such cases, clinicians may have no reliable way to differentiate between a sedated patient in a deep coma and one experiencing delirium, leading to an oversimplified and erroneous merging of these distinct syndromes. This lack of clarity distorts the conclusions of these studies and undermines their validity, as it ignores the unique mechanisms involved in the pathogenesis of each condition.

Second, the inclusion of mortality as a primary endpoint highlights another misconception of what haloperidol, or any antipsychotic, is intended to address in the context of ICU delirium. Delirium is a neuropsychiatric syndrome, not a life-threatening condition in and of itself. While delirium is associated with poor outcomes [20], such as increased morbidity and length of stay, the question of whether haloperidol, a drug designed primarily to modulate dopaminergic activity, can affect mortality is not realistic. There is no plausible mechanism by which an antipsychotic would impact the underlying causes of death in critically ill patients. Mortality in ICU patients is often the result of many other events, including severe sepsis, multi-organ failure, or other physiological insults, none of which are directly related to dopamine dysregulation [53]. Therefore, using mortality as a primary endpoint not only misrepresents haloperidol’s potential role in managing delirium but also erroneously equates the treatment of a neuropsychiatric syndrome (delirium) with the treatment of the underlying, life-threatening conditions that affect survival. By including mortality in these trials, researchers may inadvertently mislead clinicians into believing that haloperidol could have a broader therapeutic impact than it actually does. This focus on mortality as an outcome may also overshadow more relevant and pragmatic outcomes due to improved delirium management, such as reducing agitation to allow for the progression of medical care, mitigating distress and impulsive behaviors due to psychosis, or decreasing the duration of specific delirium symptoms.

When analyzing the subtypes of delirium included in the largest RCTs, another problematic finding is evident. In the 2018 study by Girard et al., 90% of the delirious patients in the haloperidol group were determined to have hypoactive delirium at the time of randomization [14]. Hypoactive delirium is characterized by lethargy, psychomotor slowing, cognitive dulling, and significantly impaired arousal [1]. In spite of this, these patients were still administered up to 20 mg of intravenous haloperidol (equivalent to 40 mg of oral haloperidol) daily. As haloperidol possesses sedative properties, this is an alarming amount to administer to patients already receiving opioids and other sedatives in the ICU and exhibiting prominent cognitive and psychomotor slowing. This is not congruent with the real-world, routine clinical management of hypoactive delirium unless psychosis and delusions are present. Even in those cases, a 20 mg daily intravenous dose remains an exceptionally high amount. Moreover, this dosage far exceeds the amount of haloperidol typically administered even to patients with other neuropsychiatric disorders (e.g., schizophrenia, bipolar disorder, dementia) for managing psychosis, mood stabilization, or agitation. This again highlights a design flaw of the RCTs and a misunderstanding of the use of haloperidol in delirium, as such high doses are not warranted in any other clinical scenario, even in well-established neuropsychiatric disorders where the evidence base for antipsychotic use is much more robust and developed [54,55,56]. The 2022 Andersen-Ranberg et al. study [15] reported a lower number of patients with hypoactive delirium (55.3%); however, they still received a median of 8.3 mg of intravenous haloperidol daily, which is still a disconcerting amount to administer for patients with this delirium subtype. As a whole, there are no theories or hypotheses suggesting that excess dopamine regulates neuropsychiatric disorders primarily characterized by cognitive or psychomotor slowing. Therefore, administering excessively large doses of haloperidol to patients with hypoactive delirium lacks a neuroscience-based justification.

Despite the major flaws of the published RCTs, there are, in fact, other very positive results that can be highlighted from these studies. The safety profile of haloperidol appears to be robust despite such high doses administered. Historically, there have been concerns about the cardiac side effects of haloperidol in hospitalized medically ill patients, particularly the incidence of prolonged QT intervals and torsades de pointes (TdP) [57]. A 2010 systematic review had reported an increased risk of these events when exceeding 10 mg of intravenous haloperidol [58]. Since then, there has been an escalation in the use of second-generation antipsychotics, given these perceived risks of haloperidol [59]. However, a recent meta-analysis of all the RCTs and secondary analysis of the 2018 Girard study demonstrated no significant increase in the QT interval or incidence of TdP [16,60]. Additionally, none of the RCTs reported any significant risks of developing extrapyramidal symptoms (EPSs) or neuroleptic malignant syndrome from using high doses of haloperidol [16]. This is intriguing, as in the psychiatric literature, haloperidol is reported to possess a higher potential to cause EPS, given its selective dopamine modulation and the putative role of dopamine in the pathophysiology of EPS [61]. As suggested by Beach et al. in their 2020 systematic review of intravenous haloperidol, this is hypothesized to be due to a baseline anticholinergic deficit in delirium pathophysiology, which may be protective against the development of EPS [13]. Finally, despite the previously mentioned issue of overemphasizing mortality as the primary endpoint, the RCTs inadvertently highlighted the most important safety aspect of haloperidol. Their results demonstrated that there was no significant difference in mortality between the haloperidol and placebo groups. This is, in fact, a positive finding, as the authors actually demonstrated that an extraordinarily high amount of haloperidol can be administered to even hypoactive delirious patients without any serious ramifications. Based on this interpretation, clinicians should feel more reassured about administering this agent in the setting of critically ill patients, as it reinforces the overall safety profile of haloperidol.

## 6. Future Directions

In order to truly advance our interventions for ICU delirium, there are a number of important considerations to revamp in future research methodologies. Most importantly, our primary endpoints and the definition of “treatment” must evolve. Rather than focusing primarily on delirium-free days and mortality, alternative outcomes deserving investigation should include the reduction in psychomotor agitation and psychotic symptoms, along with the potential clinical and functional benefits that such symptom reduction would entail. The authors of the EuRIDICE RCT astutely mentioned this, as their endpoints differed compared to other RCTs due to tracking these symptoms [44]. This would also lead to the better stratification of symptomatic treatment for different subtypes of delirium, as hypoactive delirium does not exhibit any agitation. There is also a putative role for dopamine in many types of psychomotor agitation based on the previous literature in delirium and other neuropsychiatric disorders, such as dementia, schizophrenia, and bipolar disorder [54,55,56]. Furthermore, designing clinical trials to focus on the reduction in psychosis as an outcome would be valuable, given that psychosis often leads to agitation and other negative functional outcomes [62]. This approach is supported by a wealth of data demonstrating the positive effects of haloperidol in alleviating this distressing symptom in other disorders [63]. For example, psychotic symptoms and behavioral agitation could be better assessed using established scales like the Delirium Rating Scale-Revised-98 (DRS-R-98) [64], which specifically targets delirium-related psychosis, or the Brief Psychiatric Rating Scale (BPRS), which has been widely used in other neuropsychiatric disorders, such as schizophrenia, and could be adapted for delirium-related psychosis. For agitation, the Richmond Agitation–Sedation Scale (RASS) [65], commonly used in ICU studies, is limited, as it overemphasizes motoric state and arousal instead of psychological symptoms. Overall, no single medication is likely to resolve delirium, given its complex pathophysiology. Therefore, multi-component, symptom-focused designs should be prioritized in future large-scale studies, as they more accurately reflect the clinical reality that medications like antipsychotics are typically used for symptom management and not to cure delirium itself.

Stratifying treatments for delirium based on etiology is another key area that requires further attention in ICU delirium research. The current RCTs typically involve a heterogeneous mix of ICU populations—medical, surgical, trauma, cardiovascular, and other subspecialty ICUs. Given the diverse range of critical illnesses treated in these units, the types of delirium that develop are likely equally varied [17]. Distinguishing between these different pathophysiologies based on the precipitating cause of delirium could lead to more personalized treatment strategies and potentially different trial outcomes in the future. A single agent, such as haloperidol, cannot be expected to treat all forms of delirium effectively, particularly those not driven by dopamine dysregulation. For example, the Girard et al. study [14] did not specifically exclude patients with alcohol withdrawal syndrome, a condition that can lead to delirium tremens, a hyperactive form of delirium resulting from alcohol withdrawal. The traditional treatment for delirium tremens is the use of benzodiazepines, which are positive allosteric modulators of GABA, enhancing inhibitory neurotransmission and attenuating the glutamate surge seen during withdrawal [66]. However, in other forms of ICU delirium, especially non-alcohol withdrawal-related delirium, benzodiazepines are often avoided as they can worsen delirium due to their potent inhibitory effects and anticholinergic properties [67,68]. Another important example is emergence delirium, particularly in surgical ICUs, where it is often caused by the pharmacologic effects of anesthetics used during surgery [69]. The pathophysiology of emergence delirium is not solidified but thought to involve GABA withdrawal, as anesthetics like propofol and sevoflurane are GABA modulators [70]. In this case, benzodiazepines have been cited as potentially helpful, as they can provide GABAergic support without necessarily prolonging delirium [71]. Research into short-acting benzodiazepines (e.g., remimazolam) specifically targeted for managing emergence delirium is ongoing and could offer a more tailored approach for the management of delirium in these scenarios [72].

The development of more accurate screening tools for ICU delirium should remain an important area of research, as improved detection could lead to more reliable outcomes in delirium clinical trials. The Confusion Assessment Method for the ICU (CAM-ICU) is the most commonly used tool for delirium screening and for determining outcomes like delirium-free days in haloperidol studies [73]. However, while initial validation studies reported high sensitivity and specificity (over 90%) [3,74], more recent studies using real-world, naturalistic designs have found significantly lower detection rates, with sensitivity ranging from 38% to 47% [75,76]. Notably, the sensitivity for detecting hypoactive delirium is particularly poor (31%), despite it being the most common subtype. This may be due to several factors, including limited cognitive domain testing in the CAM-ICU, lack of focus on neuropsychiatric symptoms, and an overemphasis on brevity, leading to restricted assessments. These inaccuracies in delirium detection have direct implications for clinical trials, as they could introduce substantial bias into studies that rely on CAM-ICU to define key outcomes, such as delirium-free days. To improve detection and the accuracy of outcomes, future screening tools should incorporate a broader range of psychiatric symptoms, such as agitation, perceptual disturbances, and emotional dysregulation, which are central to delirium’s core symptoms but often under-represented in many tools. The Intensive Care Delirium Screening Checklist [77] (ICDSC) and Stanford Proxy Test for Delirium [78] (S-PTD) are newer assessments that have been validated and capture a wider array of these neuropsychiatric symptoms; however, they are not yet as widely used as the CAM-ICU. Expanding the cognitive and neuropsychiatric domains assessed would allow for a more complete clinical representation of delirium, particularly in hypoactive subtypes, where subtle symptoms may otherwise go unnoticed.

In addition to enhancing detection, refining the approach to delirium subtyping and severity grading is a critical area for development. The major RCTs relied on using a concurrent RASS [65] score to differentiate between hypoactive and hyperactive delirium. This scale is characterized by a −5 to +5 grading schematic that is meant to focus on arousal and level of sedation only. Using it for subtyping is too reductionistic, as the clinical presentation of delirium involves many other symptoms and signs. For instance, diagnosing hypoactive delirium based only on whether drowsiness is present would lead to inaccurate assessments at times, as not every hypoactive patient is acutely hypoaroused. Furthermore, there are no validated guidelines for diagnosing the mixed subtypes of delirium using an RASS score. The proper subtyping of ICU delirium may allow for the stratification of different outcomes in treatment and management studies and also not propagate the concept that all types of delirium may respond to an antipsychotic such as haloperidol. Future studies should follow the Diagnostic and Statistical Manual of Mental Disorders, Fifth Edition [79] (DSM-5) or the Liptzin–Levkoff [80] criteria for adequate subtyping. The RASS score and arousal status should also not be used to conflate delirium with coma, nor should it be assumed that these two disorders always co-occur. Severity grading is also not routinely accounted for in many delirium studies, not just the recent major RCTs. This is likely due to several reasons. Firstly, although there is a plethora of screening tools for delirium, there are only two rigorously validated severity grading tools: the DRS-R-98 [64] and the Memorial Delirium Assessment Scale (MDAS) [81]. Both were created by consultation-liaison psychiatrists and are significantly more nuanced than screening tools like the CAM-ICU. As such, the DRS-R-98 and the MDAS are difficult to teach to non-psychiatrists and research assistants, given that they place more emphasis on conducting a reliable mental status examination and bedside neuropsychological tests. If severity grading was included in future clinical trial designs, this could certainly change the efficacy outcomes, as haloperidol could hypothetically reduce the severity of delirium while not necessarily decreasing the duration of delirium itself.

Although the predominant focus of large-scale RCTs has been on haloperidol, second antipsychotics (SGAs) should be investigated further as well. Most SGAs differ from first-generation agents like haloperidol primarily through their serotonin 5HT2A antagonism, which is more pronounced than dopamine D2 receptor antagonism [82]. This mechanism stimulates downstream dopamine release, leading to the less direct modulation of dopamine neurotransmission compared to first-generation agents. Additionally, the modulation of other serotonin receptors by SGAs provides mood-stabilizing effects in addition to the treatment of psychotic symptoms [83]. As such, second-generation agents are often used for bipolar mania, bipolar depression, and augmentation for the treatment of refractory unipolar depression, post-traumatic stress disorders, and anxiety disorders [84,85,86].

Several smaller-scale clinical trials have investigated SGAs for delirium-related agitation, with preliminarily positive results [9]. However, many of these trials suffer from methodologic flaws similar to those seen in haloperidol studies in terms of issues with endpoint and parameter selection. Given the positive preliminary findings, larger, more rigorous studies are needed to confirm the efficacy of SGAs in this context. Moreover, longitudinal studies would help to assess the functional outcomes of SGA use in delirium management, particularly in terms of cognitive recovery and post-delirium psychiatric and functional sequelae. It is crucial, however, to consider the potential downsides of popular SGAs in this population. For example, quetiapine and olanzapine are highly anticholinergic, which may exacerbate cholinergic deficits in delirium pathophysiology [1,87]. This anticholinergic activity could potentially lead to worsened delirium, prolonged duration, or even an increased incidence of delirium in critically ill patients [88]. Additionally, the use of SGAs with anticholinergic properties may impair cognitive recovery after a delirium episode, complicating the long-term prognosis of these patients. Overall, their role should be investigated further and compared to haloperidol in head-to-head trials, particularly for agitation and psychosis. They could also be explored in different subtypes, as their mood or anxiety augmentation effects may have a potential role in mixed or hypoactive delirium (e.g., delirium with anxiety as a symptom).

Finally, we encourage psychiatrists to become more avidly involved in ICU delirium research. A recent editorial published in the Journal of the Academy of Consultation-Liaison Psychiatry has discussed how psychiatrists have seemingly abandoned delirium research and are no longer routinely involved in major studies [89]. This is evident from reviewing the author lists of all major RCTs discussed in this manuscript, where no psychiatrist is listed as a major contributor, except in the EuRIDICE trial. Had psychiatrists been more actively involved in the design of these RCTs, the studies would likely have been better tailored to address the neuropsychiatric complexities of delirium and its outcomes, as psychiatrists are uniquely positioned as experts in the management of such disorders. Furthermore, many ICU delirium screening tools are created by non-psychiatrists, and, again, psychiatry does not appear to be significantly involved based on authorship lists. Moreover, the initial validation studies of these tools are often not compared against the gold standard, which is an assessment conducted by a psychiatrist that meets the *DSM-5* [79] (p. 5) criteria. Overall, the lack of psychiatry involvement, despite psychiatry’s historical expertise in delirium, is detrimental for the future of delirium initiatives and research. This is akin to oncologists no longer leading the charge in cancer research, despite being the ones extensively trained in this specific area.

There are likely many reasons for the limited involvement of psychiatrists in this field. However, the primary barrier appears to be the shortage of consultation-liaison psychiatrists, a specialized group who primarily work in medical hospitals. Even among consultation-liaison psychiatrists, not all are drawn to work in ICUs or focus on delirium, as the field encompasses a wide range of other diagnoses and areas of interest that attract resident physicians to pursue a fellowship in this subspecialty. To incorporate psychiatric expertise into ICU delirium research, we recommend several concrete actions. First, psychiatrists should be included as core members of multi-disciplinary research teams from the outset, ensuring that delirium is studied not just as a medical condition but as a complex neuropsychiatric syndrome. Second, there should be a push to develop specialized critical care psychiatry tracks within fellowship programs to further train psychiatrists who are well equipped to handle the unique challenges of the ICU setting. Additionally, expanding the role of psychiatrists within ICU protocols through dedicated ICU psychiatry consultation teams would ensure that delirium management includes tailored, evidence-based, neuropsychiatric interventions. Psychiatrists should also be at the forefront of improving and validating delirium screening tools, particularly by ensuring that these tools capture the broad range of neuropsychiatric symptoms, including agitation, perceptual disturbances, and emotional dysregulation, which are central to delirium’s core symptoms and pathophysiology. In summary, the increased involvement of psychiatrists in ICU delirium research is essential for advancing both the understanding and treatment of this complex syndrome.

## 7. Conclusions

Delirium remains a complex neuropsychiatric syndrome with a multifaceted pathophysiology that is not solely driven by dopamine dysregulation. A single antipsychotic or drug is unlikely to ever serve as a cure for delirium. Recent large-scale RCTs have overly emphasized the role of haloperidol in managing ICU delirium, revealing a flawed understanding of what constitutes an effective treatment for patients suffering from this condition. Instead of focusing primarily on mortality and delirium-free days, future research should prioritize the neuropsychiatric symptoms of delirium, such as agitation and psychosis, and explore the clinical and functional benefits of reducing these distressing symptoms. Future studies should also consider stratifying delirium by subtype and severity, improving detection tools, and adopting multi-intervention, multi-disciplinary approaches. Despite the fundamental flaws in the design of some published RCTs, their results highlight a robust safety profile for haloperidol in the ICU setting, with a minimal risk of cardiac or neuropsychiatric adverse events. This should be encouraging for clinicians. The methodologies of these studies echo those used in the early stages of schizophrenia research, where an overemphasis on the dopamine theory hindered advancements in treatment options. With a more modern understanding of neuroanatomy, neuroscience, and pathophysiology, we should avoid repeating such mistakes in future delirium research, ensuring a more comprehensive understanding of the condition and that our interventions are grounded in the latest scientific evidence. Moving forward, it is crucial that research efforts continue to integrate multi-disciplinary perspectives, foster innovation in delirium management, and apply rigorous, modern methodologies to improve patient outcomes in this complex and under-addressed area of care.

## Figures and Tables

**Table 1:** Review of randomized controlled trials examining haloperidol vs. placebo for delirium management.

Trial/Author Name; Year of Study; Country	Study Design	Number of Patients	Demographics	Population Characteristics	Delirium Screening Tool(s) Utilized; Screening Frequency; Motoric Subtype	Haloperidol Dosing Strategy; Amount Used	Rescue Medication Available?	Duration of Study	Primary Outcomes	**Secondary Outcomes**	**Results**	**Comments**
Andersen-Ranberg et al.; 2022; Denmark, Finland, Italy, United Kingdom [15]	Randomized, controlled trial, blinded, multicenter	987 (501 haloperidol; 486 placebo)	Median age 70; 35.3% female	Inclusion: 18 years of age or older; admitted to ICU; positive delirium screening test Exclusion: Received antipsychotic in ICU; contraindication to haloperidol; use of antipsychotic before hospital admission; delirium tremens; involuntary admission to hospital; consent unavailable; language barriers, deafness, blindness	ICDSC and CAM-ICU; twice a day; 55.3% hypoactive; 44.7% hyperactive	Intravenous haloperidol 2.5 mg three times daily with as needed dosing up to a maximum of 20 mg daily; median dose of 8.3 mg per day	Yes—propofol, BZDs, alpha-2 agonists	90 days (or discharge/death)	Number of days alive and out of the hospital at 90 days after randomization; death; length of hospital stay	Number of days alive without delirium or coma; number of days alive without mechanical ventilation; number of patients with adverse effects to haloperidol; number of patients receiving rescue medication	Primary outcome: no significant difference; secondary outcomes: no significant difference	
EuRIDICE; 2023; Netherlands [44]	Randomized, controlled trial, blinded, multicenter	132 (65 haloperidol; 67 placebo)	Mean age 64; 32% female	Inclusion: 18 years of age and older; admitted to ICU; positive delirium screening Exclusion: Admission to ICU for acute neurological condition; pregnancy or breast-feeding; contraindication to haloperidol; history of ventricular arrhythmia; neuroleptic malignant syndrome; parkinsonism; schizophrenia or other psychotic disorder; dementia or IQCODE ≥ 4; expected duration of ICU admission <24 h; language barriers, deafness, blindness	ICDSC and CAM-ICU; three times a day; 26% hypoactive; 69% mixed; 4.6% hyperactive	Intravenous haloperidol 2.5 mg three times a day, increased up to 5 mg three times a day if needed; median dose 0.08 mg/kg per day	Yes—propofol, BZDs, alpha-2 agonists, low-dose atypical antipsychotics	14 days	Number of delirium and coma-free days	Number of days with delirium, coma, or agitation; need for physical restraints; presence of hallucinations; RASS scores; mobility level; sleep quality; rescue medication for hallucinations/agitation; daily study drug dose and number of days used; self-extubation rate/removal of invasive devices; adverse effects; blood pressure after first dose of study drug; daily respiratory status; time to resolution of delirium; time to readiness for discharge from ICU; 28-day mortality	Primary outcome: no significant difference. Secondary outcomes: significantly fewer patients on haloperidol received benzodiazepines; significantly (but not clinically) lower systolic/diastolic BP after first drug dose; significantly less likely to fall/step out of bed; otherwise, no significant differences	Significant reduction in intrusive memories at discharge; also examined patient and family memories/experiences, presence of PTSD/anxiety/depression/cognitive impact, quality of life at 3 and 12 months (no significant difference)
Garg et al.; 2022; India [45]	Randomized, controlled trial, blinded, single-center	45 (15 haloperidol; 15 placebo; 15 quetiapine)	Mean age 57.32, 46.6% female	Inclusion: 18 years of age and older; admitted to ICU; positive delirium screening Exclusion: Baseline severe cognitive impairment; pregnancy or breast-feeding; neuroleptic malignant syndrome; history of torsades de pointes; contraindication to haloperidol; ongoing use of antipsychotics; rapidly resolving organ failure; moribund patients; language barriers, deafness, blindness	CAM-ICU; twice a day; subtype N/A	Oral haloperidol via nasogastric tube up to 30 mg daily; dose halved or doubled at 12 h intervals if needed	N/A	14 days	Number of days alive without delirium	Time to freedom from ventilation; time to ICU and hospital discharge; 30- and 90-day survival	Primary outcome: no significant difference; secondary outcomes: significant reduction in mean days to ICU discharge; otherwise, no significant differences	
Girard; 2018; United States [14]	Randomized, controlled trial, blinded, multicenter	566 (184 placebo; 192 haloperidol; 190 ziprasidone)	Median age 61; 44% female	Inclusion: 18 years of age or older; admitted to medical or surgical ICU with invasive or non-invasive positive pressure ventilation, vasopressors, or intra-aortic balloon pump; positive delirium screening Exclusion: Severe cognitive impairment (IQCODE ≥ 4.5); pregnancy or breast-feeding; history of torsades de pointes; QT prolongation; neuroleptic malignant syndrome; contraindication to haloperidol or ziprasidone; ongoing use of antipsychotics; moribund; rapidly resolving organ failure; incarcerated; language barriers, deafness, blindness	CAM-ICU; twice a day; 89% hypoactive; 11% hyperactive	Intravenous haloperidol 2.5 mg (or 1.25 mg if 70 or above) every 12 h. Dose doubled if remained delirious; halved if no delirium. Maximum 20 mg/day; median dose of haloperidol 11 mg ± 4.8 mg per day	Yes—open-label antipsychotics	14 days (or ICU discharge)	Number of days alive without delirium or coma	Duration of delirium, time to freedom from ventilation, ICU discharge, ICU readmission, hospital discharge, 30/90-day survival, adverse effects	Primary outcomes: no significant difference; secondary outcomes: no significant difference	
ORIC-I; 2017 (study terminated); United States [43]	Randomized, controlled trial, blinded, single-center	Trial terminated due to insufficient recruitment	N/A	Inclusion: 18 years of age and older; admitted to medical, surgical, trauma, or cardiothoracic ICU; mechanical ventilation; positive delirium screening Exclusion: History of schizophrenia or neurologic disease that may confound the delirium assessment; QT prolongation; Parkinson’s disease; pregnancy or breast-feeding; extubation prior to enrollment; treatment with haloperidol within 2 days of ICU admission; incarceration; language barriers, deafness, blindness	N/A	Intravenous haloperidol 5 mg every 12 h	N/A	N/A	28-day mortality; 90-day mortality	Duration of mechanical ventilation; ICU length of stay	N/A	

Abbreviations: BZDs = Benzodiazepines; CAM-ICU = Confusion Assessment Method for the ICU; ICDSC = Intensive Care Delirium Screening Checklist; IQCODE = Informant Questionnaire on Cognitive Decline; PTSD = Post-Traumatic Stress Disorder; RASS = Richmond Agitation–Sedation Scale.
